# “Where was this when I was in Physical Education?” Physical literacy enriched pedagogy in a quality physical education context

**DOI:** 10.3389/fspor.2023.1185680

**Published:** 2023-05-25

**Authors:** Natalie Houser, Dean Kriellaars

**Affiliations:** College of Rehabilitation Sciences, University of Manitoba, Winnipeg, MB, Canada

**Keywords:** schools, children and youth, teachers, students, physical activity

## Abstract

**Introduction:**

In recent years, there has been a call to restructure physical education (PE) practices and outcomes. A physical literacy enriched pedagogy approach would support this change by more intentional design of lesson planning that includes concurrent development of competence & confidence and inclusion of students of all levels of ability, leading to holistic development of the student. Despite this potential, there is little research to date that outlines PE pedagogical practices with physical literacy as a foundation. The purpose was to explore pedagogical practices and perspectives from elementary PE teachers through a physical literacy enriched pedagogy lens in a high-quality PE context.

**Methods:**

One-on-one semi-structured interviews were conducted with a convenience sample of elementary PE teachers within one school division. Interviews with all participants focused on questions related to PE and physical literacy. Thematic analysis was used to analyze the data collected from the audio-recorded interviews.

**Results:**

Four themes were generated based on the semi-structured interviews from six elementary PE teachers from one school division. The results identified key physical literacy enriched pedagogical practices based on four themes: supporting a holistic PE experience based upon physical literacy as an outcome; movement within and beyond PE; inclusive and individualized experiences; and physical literacy practices bringing the school community together. The findings were then connected to the physical literacy cycle and UNESCO components of quality PE.

**Conclusions:**

All participants spoke to how their pedagogy focused on the holistic development and inclusion of their students based upon activation of various feedback pathways of the physical literacy cycle. The themes that emerged and subsequent insight gained from teachers went beyond existing physical literacy cycles, in particular by discussing development of students from cognitive, affective, social and creative (problem solving) perspectives, supporting an expansion to the existing physical literacy cycle as presented.

## 1. Introduction

Despite extensive knowledge on the importance of movement, and the negative downstream effects of insufficient activity (1), few sustainable solutions exist in today's movement suppressed culture, which has only been further exacerbated as a result of the COVID-19 pandemic (2). With a physical activity approach focused on health outcomes (i.e., meet the guidelines to avoid non-communicable disease), little attention is directed at why we move, and the motivational structures associated with this. Though there exist various approaches to increase physical activity, few long-term successes (limited to efficacy) can be demonstrated (3, 4), and part of this failure may be due to a focus on instrumental valuation as opposed to intrinsic valuation of movement (5). Shifting away from the dehumanizing physical activity promotion strategies to support intrinsic valuation of movement (6), as exemplified in physical literacy through concurrent development of competence and psychological characteristics such as confidence and motivation (7), may be a way to support more sustainable behaviour change. One of the contexts in which this shift in thinking may be particularly impactful is in schools where positive movement experiences can be supported (8, 9).

As identified by the United Nations Educational, Scientific and Cultural Organization (UNESCO), physical education (PE) is critical to school community health and physical activity (10, 11). Specifically emphasized by UNESCO is the idea of quality physical education (QPE), of which the outcome is physical literacy (10, 11). In recent years, there has been a call to restructure PE practices and outcomes (12–14) which necessarily includes a disruption of the traditional sport-based PE model (15). Many PE curricula now include statements around physical literacy (e.g., 16), but it is also critical to provide effective teacher training to support holistic physical literacy enriched approaches (17). Meaningful PE is a requisite part of QPE (18), and QPE necessarily involves the adoption of a physical literacy informed and enriched pedagogy. Physical literacy enriched pedagogy has broad applicability to movement contexts, resulting in more intentional design of lesson planning that includes confidence building [e.g., development of a sense of pride (19) and opportunities to exercise agency (20, 21)] through the construction of positive challenge (22). A physical literacy enriched approach would also allow for a greater level of inclusion in lesson plans, increased capacity to retain and progress participants, and a shift away from a solely technical movement focus to a holistic approach that includes psychological and social aspects related to a child's movement experience (23). Physical literacy enriched pedagogy in the PE context may provide a unique way to activate the physical literacy cycle for all students which would necessarily subsume non-linear pedagogy (24) leading teachers to be deliberate in the way they deliver instruction and set up their space for learning.

Although activation of the physical literacy cycle in the PE context is critical, to date there is little evidence that outlines the pedagogical practices using physical literacy as a foundation, that would support a holistic approach to fostering movement potential for all students. Therefore, the purpose was to explore pedagogical practices and perspectives from elementary PE teachers through a physical literacy enriched pedagogy lens in a high-quality PE context.

## 2. Methods

### 2.1. Study design

A qualitative interpretive description study design (25) was employed to gain a better understanding of PE teacher pedagogical practices. This approach is ideal in the school context as it provides a research process that supports enacting meaningful results within applied disciplines (25). The primary method of data collection was one-on-one semi-structured interviews. Ethical approval was obtained from the University of Manitoba's Health Research Ethics Board (H2021:401) and divisional approval was also granted.

### 2.2. Context and participants

The PE context in Manitoba is unique relative to other places in Canada and internationally as there is mandatory PE for kindergarten through grade 12, with PE 3–5 days per week (30–60 min/class), and nearly all teachers delivering the PE curriculum are specialists. Class sizes are small and nominally set to 25 with a range of 20–30 students per class. Since 2009, province-wide PE has largely been focused on the development of a child's physical and health literacy.

The elementary schools from a single school division were identified as a convenience sample based on the conditions and longstanding quality initiatives that have been undertaken to foster quality PE. These initiatives and conditions include; for over a decade, an average of 4 full days per year of PE and physical literacy focused professional development for PE teachers; the existence of an active community of practice using smartphone applications (e.g., WhatsApp, Instagram) and cloud-based document sharing for progressions; the existence of a full time PE/HE coordinator that provides support for the 15 elementary schools in this division including acting as an advocate to the school board and trustees for funding, grant writing, liaison to other school staff and administrators.

Six elementary (K-5) school PE specialists within this division were recruited based on a general recruitment email sent to the 15 elementary schools in the division. The PE classes were nominally 38 min in duration with a frequency of 4–5 classes per week. All participants have previously attended physical literacy focused professional development, and three attended the circus instructor training program at the National Circus School. The six participating teachers (5 male, 1 female) ranged from 2 years to 15+ years of teaching experience, all teaching kindergarten through grade 5 PE, within a school division serving low to middle socioeconomic families.

### 2.3. Procedure

PE teachers were invited to discuss their experiences teaching PE in an audio-recorded interview lasting 45–60 min. Interviews with all participants followed a semi-structured interview format, focusing on questions related to physical education and physical literacy. The semi-structured interview guide was developed through consultation with a co-author and with the PE/HE coordinator in the division. This co-production process is referred to as “equitable and experientially informed research” by Smith et al. (26). Interviews took place virtually in the Spring of 2022. Upon completion of the interviews, the first author transcribed all audio recordings verbatim. Participant quotations will be included in the results section and include participant pseudonyms.

### 2.4. Analysis

Data analysis followed a six-step thematic analysis process which included familiarization with the data, generating codes, identifying themes, reviewing themes, defining themes, and creating the report of results (27, 28). Codes were generated for each transcript, and the themes were then cross-checked by a co-author (i.e., critical friend), as suggested by Gibbs (29) to confirm the codes were generally agreed upon by another researcher. Data trustworthiness was established based on aspects of credibility, transferability, and dependability (30).

**Figure 1:**
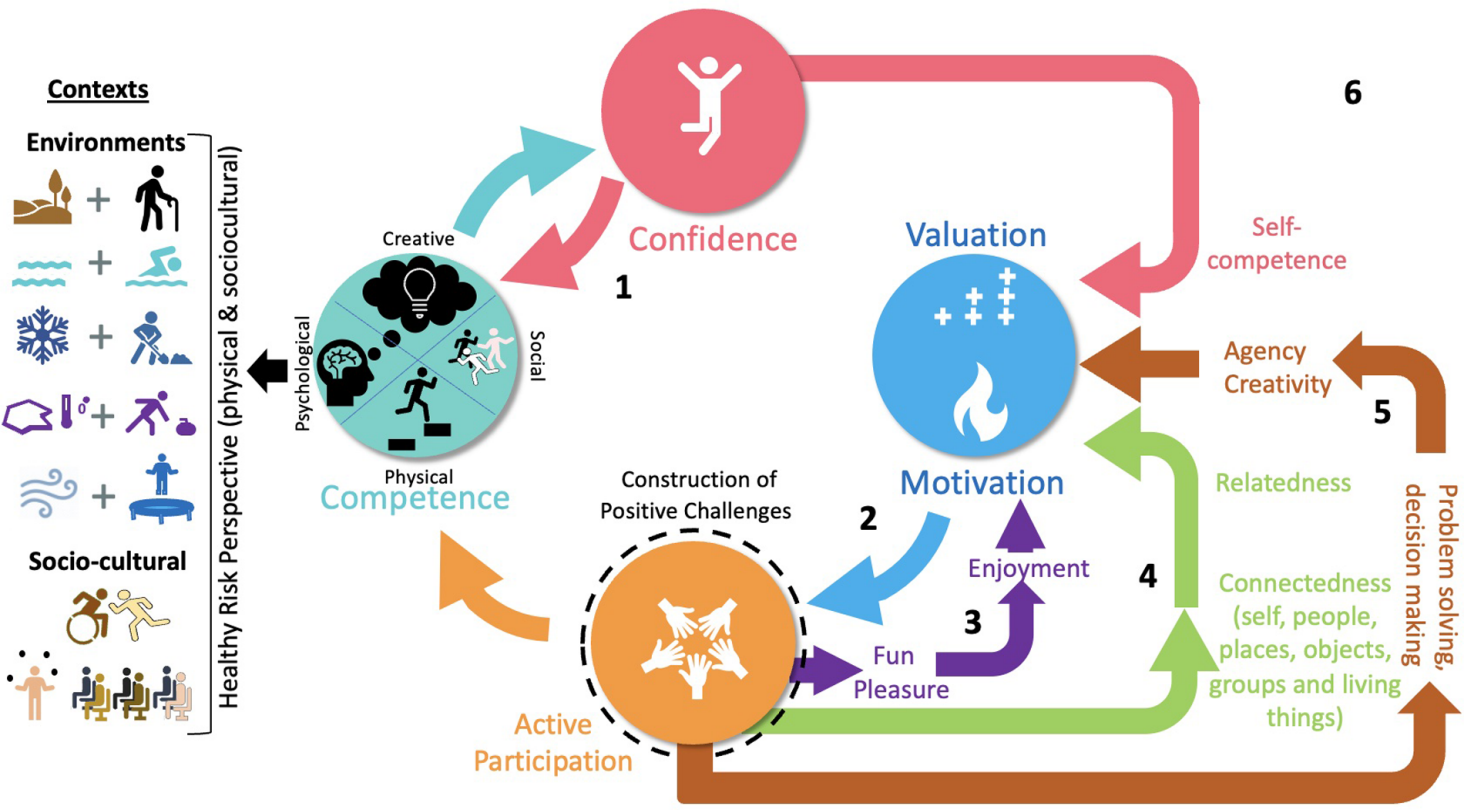
Enhanced physical literacy cycle, modified from Stuckey et al. (31). Feedback pathways: (1) competence-confidence; (2) motivation-active participation; (3) fun-enjoyment-pleasure; (4) connectedness-relatedness; (5) creativity-agency; (6) general cycle related or positive movement experiences.

Upon scrutiny of the transcribed data, there was consensus by both authors that the thematic elements and related quotations could be readily attached to the feedback pathways of the physical literacy cycle [shown in Figure 1 and derived from Stuckey et al. (31)] especially since it is a desired outcome of QPE (11). Further, we connected the findings to components of QPE, as outlined by the UNESCO statement: “Quality physical education is distinct from physical education. The main differences relate to ***frequency, variety, inclusivity*** and ***value content***. Quality physical education is about ***peer-led learning*** and ***rounded skill development*** which can enhance educational and employability outcomes” (11).

## 3. Results

Four major themes were generated to summarize the participants' pedagogical practices: (1) *Supporting a holistic PE experience based upon physical literacy as an outcome*, (2) *Movement within and beyond PE*, (3) *Inclusive and individualized experiences*, and (4) *Physical literacy practices bringing the school community together*. Further, quotations within each theme were attached to activation of one (or more) of the feedback pathways of an evolved physical literacy cycle shown in Figure 1: (1) competence-confidence; (2) motivation-active participation; (3) fun-enjoyment-pleasure; (4) connectedness-relatedness; (5) creativity-agency; or labelled as (6) being generally related to the physical literacy cycle (31) or generating a positive movement experience (32). Quotes were also labelled by theme number (T) and quote number (Q) within that theme for linkages to QPE components.

### 3.1. Theme one—supporting a holistic PE experience based upon physical literacy as an outcome

There was strong support for physical literacy enriched approaches among all teachers, and aspects of physical literacy were identified by all participants as the goal of their PE class, as discussed by Lenny:

“I truly believe in the idea of physical literacy, getting the kids the skills and confidence to try things on their own. I think if we can get kids to be confident, competent movers, then we've sort of hit our goal.”- Lenny (*1,2*)- T1Q1

In fact, participants described their role as a PE teacher to include exposure to and experiences in a wide variety of different activities, departing from the traditional sport exposure model. The teachers focused on PE as a vehicle for students finding their movement interests, ones that the student could use for the rest of their life, which is nicely described by Eddie as: “opportunities to get engaged in a variety of different activities to improve their physical literacy” (*2*)-T1Q2. This role as a PE teacher is further supported by Marley who describes PE as an embodied movement experience:

“My biggest role is to teach children or facilitate experiences where they get to know their body and how it moves in the world and the importance of just using their body so that they will have health and wellness as they move forward the rest of their lives.”- Marley (*1,2,4*)- T1Q3

In their own way, all participants described teaching strategies that supported activation of all components of the physical literacy core cycle (competence→ confidence→ motivation→ active participation) (33). Within the competency domain of physical literacy, Eddie discusses the importance of finding a level of challenge for all levels of ability so as to engage all in a competency progression, and incrementally progress each student:

“I think just knowing how to progress the students, where to start with the students for the proper grade level and age level, I think that's one. And then being able to recognize when it's time to move on to new progressions and what progressions to use, what aims to help them, to teach them, or to introduce to them, to keep them engaged, keep them learning and allow them to practice and apply their skills in.”- Eddie (*2,5*)- T1Q4

In addition to competency progressions, teachers also referred to the importance of enjoyment and confidence development in their students. Lenny mentions their views on the critical and intentional linkage between enjoyment and confidence for holistic development of the student:

“I think the biggest thing for me is trying to get them to enjoy the things that they're doing so that they have that confidence. Because at the end of the day, with the age I'm working with, they're still kids. So, if they're not having fun, then they're not going to do these sorts of activities when they're older. And that comes with confidence. So just giving them the tools to learn things that they're able to do and also have a variation of activity”- Lenny (*1,2,3*)- T1Q5

Though discussed less explicitly, participants often referred to student motivation in alignment with engaging all the students in active participation. Marley discusses how frustration as a result of striving for perfection sometimes results in demotivation to participate, but also highlights strategies used to mitigate this:

“I have very few instances where students are sitting out or feeling overly frustrated. It still happens, but mostly because they're at the point they're in the mind frame where they have to do this skill the perfect way. But for people that are invited to just express themselves in different ways to like I have very few people feeling less confident with their skills, I think.”- Marley (*1,2,5*)- T1Q6

In order to obtain engagement of all students of different abilities, Gord discusses strategies to avoid social inhibition (the so-called audience effect) among their students through small group activities:

“But overall, it's definitely a way to increase participation. Those kids that maybe would shy away are now a little bit more keen to come do something in the day like. So if we're working on just some coordination and agility type skills with ladders and obstacles and stuff like that, if they're an uncoordinated kid, they're going to maybe shy away from that. But if it's just a small group and a couple of kids, they'll definitely I feel like they're more keen to come and try it out there.”- Gord (*1,2,4*)- T1Q7

### 3.2. Theme two—movement within and beyond PE

Participants listed a remarkable number of movement opportunities that students are exposed to within this school division, that span well beyond the traditional sport-centric focus in PE. Some unique activity examples that the teachers provided included: “wheeled motion”, fat tire biking in snow, bouldering/rock climbing, the key families of circus arts (acrobatics, manipulation, equilibrium, and aerials), parkour, cross country skiing, disc golf, Indigenous dance, swim programs, and fishing/ice fishing to name a few. Teachers understand the value of movement in diverse physical and sociocultural contexts, and all emphasized the importance of movement experiences beyond the school grounds and within their community. As the teachers listed the different activities their students were exposed to, they expressed the importance of these exposures for future participation:

“I believe my role is to be part of positive experiences and connections with movement, help kids build confidence so that they can further their competence. That is language that's been in my mind lately going to pursue lifelong physical activity so that they're comfortable moving in different domains.”- John (*1,2,4*)- T2Q1

Despite the pressure often placed on PE as a way for students to achieve minutes of physical activity, no teachers in this study mentioned achievement of moderate or vigorous activity as the goal of their PE class or that becoming a “sweaty mess” was the primary experience they were hoping for students to get out of PE. This shift away from a physical activity way of thinking to a physical literacy way is supported by Gord:

“That's my hope is that they're going to go out and do it on the weekend because that's the problem, they come to school 9 to 4, and then they go home and plop down on the couch and I'm like, okay, that defeats the purpose, right? Like, okay, it's good you're active then, but now you're kind of giving up, right?”- Gord (*2*)- T2Q2

In addition to supporting lifelong active participation through the content delivered in PE, once again confidence and competence in a range of different activities is mentioned by Eddie as a way to foster both a lifelong interest in movement and an ability to self-organize (agency): “We had a girl who learned to ride the unicycle. Her mom went out and bought her unicycle. Now she's riding unicycle down the sidewalk at her house.”- Eddie (*1,5*)- T2Q3

As the interviewed teachers are often trying new activities with their students, a sense of trust and communication was identified as important between the teacher and their students, and perhaps the teachers were establishing a more effective working alliance through tripartite efficacy (34). As such, rapport with students was also mentioned by Eddie as a critical part to success in the gymnasium:

“I think we have a good relationship and a good rapport with each other. So I think we are able to respect one another and we have that classroom management that is a lot better. Just because they want to be in the gym. You spend a lot of time in the gym working together on skills. You're helping them to build skills and build confidence. And I think all that allows you to build a good relationship with a student and a good rapport.”- Eddie (*1,4*)- T2Q4

### 3.3. Theme three—inclusive and individualized experiences

Physical literacy as a construct that is inclusive by design, as suggested in the core principles of Canada's physical literacy consensus statement (35), was supported by many of the participants in terms of their practices and experiences. Gord shares how their classes are structured “so those that do have any kind of intellectual disability or physical disabilities are coming in and I feel like they're there in the program, just like everybody else, there is not really any differentiation happening” (*2,4*)- T3Q1. This approach is further supported by John, where they discuss how they strive to have everyone authentically participating:

“Yeah, I think any time you can widen that reach so that everyone is participating in the same activity, I think that you're achieving inclusion because you're not pushing anyone to the side necessarily. You're saying, okay, we're working on balance, we're working on coordination, we're working on strength, we're working all these areas of success. Someone's working on balance. They're included just as much as the one up in the air working on strength. They're all part of the same production.”- John (*1,2,6*)- T3Q2

Participants also discussed the importance of individualized approaches in class to further foster the development of a child's physical literacy regardless of a student's level of ability. Specifically, Gord mentions a student peer to peer teaching strategy:

“So I feel that, that idea of the station work and the individual work and that peer teaching, I think it makes it a very welcoming environment to anybody, whether you're skilled or not skilled or you have any kind of disability that's affecting how you might participate in phys ed. I don't think I don't see a lot of exclusion in any way in our program, it's very inclusive to everybody.”- Gord (*4,6*)- T3Q3

The peer teaching also saw spillover into the recess space, as further described by Gord: “In terms of recess activities, the peer teaching, you see it outside. They'll be doing games outside, they're playing and there will be kids teaching other kids how to do certain skills” (*4,5,6*)- T3Q4. Some of the traditional sporting games (TSGs) commonly played at recess included tag games, ball games, and team games. The wide range of physical, psychological, and social abilities within a single PE class can make inclusive practices challenging at times, but John discusses how they make it work, and the benefits when it is accomplished:

“I think the first thing that jumps to mind directly relating to circus is the ability to differentiate instruction. It's by far the hardest thing to do. When you see the wide range of abilities that we work with in a class of 25 kids, that being able to reach all sides, that umbrella or that spectrum of learner… I think circus just kind of naturally creates that umbrella in that safe space for students to step into that world. On whatever scale they feel comfortable with, whether they're just wanting to stay grounded, try something low risk, but at the same time challenging. They can build from there.”- John (*1,2,5*)- T3Q5

Further supporting the idea of an umbrella of safe space, by providing students with individualized challenges, teachers also recognized the positive impact on student resilience. Eddie shares how they teach students to deal with failure, and the fact that failure is not the opposite of success, but a part of it:

“That's that resilient piece, right? Like learning to fail. You're going to get scored on. What do you do? Or you're going to drop a ball in juggling or, maybe you can’t do a cartwheel at this time, but let's work on these progressions and eventually you'll get there.” – Eddie (*1,2*)- T3Q6

These individualized approaches also include opportunities for creativity through movement, as described by Marley: “It's more of a global movement of like, how do you like to move and just and ways that we can get there faster, or ways that we could get there slower, and ways we could get there higher, and we get there lower” (*2,6*)- T3Q7.

Teachers also described how their risk perspective shifted from a somewhat surplus safety to an adequate safety mindset (36) based on their experiences and professional development. As exemplified by Gord: “I think post circus training, my tolerance for risk went up like 100-fold” (*1*)- T3Q8. This teacher's mindset fostered a healthy risk perspective in their students “if you feel safe doing it and you can do it, then do it” (*1*)- T3Q9 for physical contexts of risk but this mindset also fosters a healthy psychological risk perspective through student peer mentoring and performance. This shift in risk perspective by teachers then transferred to how they allowed their students to manage risk, find their optimal challenge, and thus create their individualized experience. Gord elaborates on this by stating:

“I like the idea of if you feel comfortable doing it and you feel safe, then do it. And I feel like giving that ability to the individual, it's unreal. On some of the aerial stuff we do, like if you tell the kid, if you feel safe, you want to try it and you know how to fall safely and then they're the best judge of their own ability versus me saying, ‘everyone's doing back rollovers’. Some kids will never do that because they're too afraid or some kids will do like, can I do it off a box? They're making up these crazy skills so they're the best indicator of their own success, their own challenge level.”- Gord (*1,2,5*)- T3Q10

### 3.4. Theme four—physical literacy practices bringing the school community together

The participating teachers have described the various ways in which physical literacy enriched practices are present, but they have also noted how the physical literacy language has helped as well. Marley mentions “yes, it's helpful to have that language to kind of express my beliefs that I kind of have that have developed over time” (*6*)- T4Q1. In addition to language that supports their practices, participants emphasized how their physical literacy enriched PE garnered attention from parents:

“I hear a common response from a lot of parents and teachers is this isn't what it looked like for me. I wish it looked this way. I wish this was the kind of phys ed classes that I had or the experience I had because I probably would be or would have been more likely to pursue movement.”- John (*6*)- T4Q2

Gord further emphasizes the connection with parents, while also mentioning the unique collaborations that circus in PE allowed for among PE, music, and drama teachers. This remark was shared by the other participants who also run a circus arts instruction program.

“When parents have been in the building over the last, let's say five to six years, then yeah, for sure, you get a lot of the comments like ‘oh where was this when I was in phys ed’? You always hear that comment. And then, when circus first came in it was a big, big hoopla for the parents. They were like, this is amazing. So, we had a circus performance one night and the next year we tied in with our music teacher, did a circus themed musical. So that was a really big deal when it first came in and still is big, but it's not quite the same when it first started up for sure.”- Gord (*1,4,6*)- T4Q3

A supportive school-community environment is also key when teachers are trying new things in PE, as identified by Lenny:

“I work at a school where everybody's really supportive, so that made it really easy for me to like to know that if I tried something and it didn't work, I wasn't going to get in trouble, there wasn't any real huge consequences to that. So, giving that like carte blanche to really give those things a try was really important.”- Lenny (*1,4*)- T4Q4

Additionally, school support staff play an integral role in the physical education experience of students, and John highlights how their thinking has evolved around working with support staff, and the effort it sometimes took to get there, given previous negative experiences of the support staff:

“The percentage is shocking, how many people did not have a very good experience with their phys ed class or with their teacher. I think that it opens their eyes not just for them personally, but for how they perceive children who are struggling in the class academically and then they see them succeeding in the gym. I think it builds a feeling of hope within physical education… I think that when we get language that's shared amongst staff and that's happening a lot in my building, with support staff and teachers being educated on what we're working on to create an inclusive space. There's a shared common goal that's really valuable that gives a feeling of optimism to the kids.”- John (*2,4,6*)- T4Q5

Another key piece is the community of practice that has developed among teachers within the division. Eddie describes the importance of the communication that takes place between teachers as a way to further develop their thinking and pedagogy around physical literacy:

“We're a pretty good group. And amongst all the early years teachers in our division, we have kind of our own, like not our own little groups, but we have kind of the circus group that we all talk to. And I'd say I give credit to the PE/HE coordinator again about giving us release time and opportunity to get together to create documents on what's the best circus progressions for the new upcoming teachers or teachers who are taking the circus course and starting out. I give credit to the PE/HE coordinator for allowing us to have time to get together and talk about it and make documents on the kind of the trial and errors that we've had. And what we figured out was the best progressions to deliver the circus program or the teaching games for understanding program. Yeah. I think, there's definitely a strong community.”- Eddie (*1,2,5*)- T4Q6

The community of practice that Eddie mentions, in addition to the collaborations with other teachers and administrators within an individual school, and connections with parents presents an encouraging community developing around physical literacy enriched practices.

### 3.5. Connecting the findings to QPE

UNESCO (11) identified six elements as foundational for QPE leading to the development of physically literate students: frequency, variety, inclusivity, value context, peer-led learning and rounded skill development. All six of these QPE components were supported by findings in this study. *Variety* was exemplified by teachers not only by the number of different activities that students were exposed to, but also regarding variety of teaching approaches and class styles (e.g., circuits, self-directed/peer-led, teacher-led, etc.) (see T1Q2, T2Q4, T3Q3). *Inclusivity* was demonstrated based on the provision of a level of challenge for all levels of ability (see T1Q4) and variety of activity disciplines offered which reduced potential biases related to ability or gender (see T3Q1, T3Q2, T4Q5). Although inclusivity was not a topic that was specifically probed in the interviews, many teachers spoke to having children find their passion for movement or “movement voice”. The teachers adopted numerous approaches to achieve positive valuation (*value content*) of the PE class by the students. These included the provision of a variety of content and options that would be perceived as relevant and activating for the child. Further, teachers identified that a goal was to have all children achieve a net positive emotional valance to participation in PE class (37) leading to a sense of belonging (see T2Q3, T4Q1). *Peer-led learning* was a strategy often discussed as a means to further engage and support positive experiences for all students (see T3Q3, T3Q4). *Rounded skill development* was evident based on teachers identifying the importance of providing a variety of movement experiences beyond the traditional sport-centric approaches (see T1Q2, T2Q1), and the emphasis all teachers placed on preparing students for continued movement beyond the school context (see T1Q3, T2Q2, T3Q7). Although there are no specific quotes for *frequency*, the delivery of PE met or exceeded the minimum standard for elementary schools in Manitoba (target of 150 min) with a frequency of 4–5 classes per week and class durations of 38 min. Interestingly, this school division also had a high frequency of professional development days offered per year (about 4 per year), as well as a strong community of practice, which was discussed (see T4Q6).

### 3.6. Enhanced physical literacy cycle

Based upon the participants' discourse and explanatory to the themes, the physical literacy cycle (See Figure 1) proposed by Stuckey et al. (31) was modified to; include four sub-domains (physical, psychological, creative and social) in the confidence-competence pathway to represent the holistic development of the student; include different forms of connection (self, people, places, objects, groups and living things); explicitly include a decision making/problem solving feedback pathway leading to agency and creativity (replacing autonomy); include the notions of fun and pleasure leading to enjoyment of movement; and the notion of developing a healthy risk perspective being developed across physical and psychosocial contexts.

## 4. Discussion

This study identified the practices and perspectives from six elementary PE teachers through a physical literacy enriched pedagogy lens in a high-quality PE context. Four themes emerged that provided a better understanding of physical literacy enriched pedagogy. The themes from the interviews predominantly identified processes based on their practices, and we saw a natural fit to the most recent iteration of the physical literacy cycle (31). All participants spoke about how their pedagogy focused on the holistic development of their students, which emphasized activation of different feedback pathways of the physical literacy cycle (31). The themes that emerged and subsequent insight gained from teachers went beyond existing physical literacy cycles (31, 33), in particular by discussing holistic development of students from a physical, psychological (cognitive and affective), social and creative (problem solving) perspective, supporting an expansion to the existing model as presented in this paper (Figure 1). The holistic nature of participants' practices supports the idea that these PE programs were representative of QPE, which includes: “Quality physical education is distinct from physical education. The main differences relate to frequency, variety, inclusivity and value content. Quality physical education is about peer-led learning and rounded skill development which can enhance educational and employability outcomes.” (11). The unique characteristics mentioned by UNESCO, which similarly align with features of meaningful pedagogy (18), were evident in the participants' description of their teaching practices, supporting a QPE experience and physical literacy development. The physical literacy enriched pedagogy of these teachers were well suited, and in fact directed to development of a physically literate student which included consideration for the emotional wellbeing of participants with all levels of ability. Further, the pedagogy fostered the creation of creative agency through the provision of problem solving and decision-making practices (as opposed to purely prescriptive approach).

Physical literacy enriched pedagogy can support new ways to engage students in a positive movement experience in PE through achievement of a net positive emotional valence (37), and perhaps prevent the accumulation of negative movement experiences (32). Physical literacy enriched pedagogy practices outlined by teachers align with existing literature and included: a level of challenge for all levels of ability (22); genuine connection (to self, people, places, objects, groups, and other living things) (22) leading to the development of a movement voice and a sense of belonging; teamwork and empathy (38, 39); opportunities for creative movement (40); the development of a healthy risk perspective (36); concurrent confidence and competence progressions (31); and a high level of engagement by all (active participation) (8). More specifically, teachers mentioned how their pedagogy focused on competency development, in the form of physical competence, but also social, creative and psychological competencies such as peer to peer teaching, prescriptive and creative progressions, and mitigating student fear of failure (i.e., intentional confidence development). Integrating progressions, which was mentioned by all participants, further supports intentional confidence development as it offers a level of challenge for all levels of ability (physical, social, creative, psychological) (22). Teachers identified the importance of setting the optimal challenge for each student, where the positive affect accomplished during and upon completion of tasks would supersede the initial negative emotions, such as anxiety or self-doubt. Students engaged in “overcoming challenges” may be a resilience building process leading to enhanced ability to overcome adversity in and beyond the school walls (33). The valuation of movement in different contexts as exemplified by these teachers, may provide an affordances mindset for students to act as their own agents within and beyond the PE context (20, 41). The participants identified the importance of authentic relationships with students, this could foster a working alliance through the development of tripartite agency (34) leading to positive valuation of the PE classes (37). Children have identified that being engaged in a competency progression (i.e., learning something new) is fun (42) and upon reflection these children would associate enjoyment with the class leading to increased likelihood of lifelong participation in movement (14, 43). Through the intentional construction of positive challenges using physical literacy in PE, participants also suggested this supported student engagement, motivation, and continued participation within their PE classes. Ultimately, given repeated provision of positive movement experiences in the PE context, this could lead to the association of happiness with physical activity as students emerge into adulthood (9, 32). The combination of these pedagogical practices over the course of a PE program supports the activation of the physical literacy cycle and the various feedback pathways, in a variety of different movement and social contexts.

Despite the emphasis of PE as a way for students to achieve minutes of MVPA (44, 45), no teachers in this study mentioned MVPA as the goal of their PE class or as the primary outcome they were hoping for students to get out of PE. Although it is agreed that a more active society is important, if physical literacy is not the foundation for pedagogy, it is likely practice will revert to a physical activity focused “sweaty mess” thinking. While physical activity and fitness motifs were not the goal, as a result of the practices and high levels of student engagement, these outcomes were likely still achieved secondarily. This finding is in alignment with Ramer and colleagues who found that enjoyment of physical activity was a better predictor of future participation than the amount of MVPA (43). Teachers' strategies were focused on more holistic student development and future activity participation, not typical sport-centric approaches (15) including intentional play and performing arts-based exploration of skill development. This demonstrates a shift in thinking among participating teachers towards intrinsic valuation of movement as opposed to instrumental valuation of movement focused on “meeting the guidelines” and fitness motifs (5, 6). Many of the teachers discussed how their goal is to expose students to a wide range of different activities (in different environments), to support continued participation in activities of their choosing within and beyond the school context, emphasizing student inclusion and an ability to self-organize movement (agency) fostered through decision making opportunities. As reported by Bremer et al., children highly value movement, and when children have the opportunity for creativity and agency, the physical literacy cycle is further supported (22, 40, 41). An example of the inclusive and agency supportive environments in these PE classes is “wheeled motion”, where students can choose to navigate the constructed gymnasium space using the wheeled device of their choosing (e.g., scooter, razor bike, rollerblades, ripstik, etc.). Another example mentioned by the participants was the shift away from traditional climbing approaches to the creation of bouldering walls with the intent of teaching students how to navigate the world and problem solve, rather than achieve specific climbing levels. In this way, students once again were provided with choice requiring decision making, and the different levels of challenge provided were conducive to all participants finding a competency challenge suitable to their level of ability (21, 46, 47).

The physical literacy enriched delivery of TSGs has been identified as a critical component of quality pedagogy by participating teachers to support holistic development of their students through quality PE practices (11). For example, physical literacy enriched “teaching games for understanding” games, which include target games, striking games, net/wall games and invasion/territorial games (48), were identified as key elements for PE curricular delivery. Physical literacy enriched pedagogy in combination with nonlinear pedagogy, can be used to guide the development and adaptation of all forms of games, including TSGs, for the achievement of inclusive and positive movement experiences (22, 49). Further, the approaches outlined in this paper within the PE context have immediate application to support TSGs and other games in recreation, performance arts, early childcare, and afterschool sport programs, which has been described in other studies (50).

This may be a model jurisdiction QPE in practice. There is clear value placed on physical literacy and quality PE within this division based on their multiple professional development days a year focused on physical literacy and quality PE, a designated PE/HE coordinator (who fosters and facilitates 3–5 professional development days per year, as well as performed organizational and acquisitional duties that would normally be on the “side of the desk” of teacher), and strong support from administration (superintendent, trustees, and city councillors), other staff, and parents. The combination of these investments and efforts has resulted in the intentional design of PE opportunities that support physical literacy development of all students (holistic and inclusive). Effective delivery of curricular outcomes is dependent on the type of pedagogy illustrated here, and systems to foster this pedagogy are required (education degree, professional development, etc.) to further the quality physical education initiative (10, 11). Further, the physical literacy enriched pedagogy that has emerged in this PE context would have clear application in fostering quality coaching, recreation and even performing arts pedagogy. We believe that a combination of the above factors in other jurisdictions would be a promising way to support physical literacy enriched pedagogy leading to a jurisdiction which exemplifies QPE.

### 4.1. Limitations

The current study is limited to the province of Manitoba, and although many experiences may be similar in other provinces and countries, the potential for variation in supports and priorities exist. For this reason, there is an opportunity to conduct future research in implementation science in different communities, to explore the similarities and differences in experiences, looking at process and outcomes. Given the potential differences based on location, future research could expand on the importance of clear roles and priorities within a physical literacy enriched pedagogical approach in different regions. This study was also delimited to the elementary context, and caution is advised to extrapolating these findings to middle and high school contexts, until further research is conducted.

## 5. Conclusion

The four major themes presented here provide support for the notion of physical literacy enriched pedagogy based upon activation of the physical literacy cycle and addressing all the key components of QPE as proposed by UNESCO (10, 11). The inclusive pedagogical practices articulated here support the holistic development of students including their socioemotional wellbeing and their agency and creativity (intelligent decision making). This school division has taken many steps to develop a supportive physical literacy and PE co-culture, providing a quality example of physical literacy enriched pedagogy in practice. These steps have led to teachers seeing merit in using physical literacy as a vehicle for QPE. There was a clear articulation by the participants of activating various feedback pathways of the physical literacy cycle directed to the development of movement skills but also the development of agency through problem solving and decision making; intentional and concurrent development of competence with confidence, a key psychological characteristic for wellbeing; provision of a level of challenge for all levels of ability for inclusion; and fostering various forms of connection for the student's belonging through movement. Insights provided by the teachers allowed evolution of the physical literacy cycle. This physical literacy cycle could be used as a framework for lesson planning in PE, as well as other movement contexts (sport, recreation, performing arts) for holistic development of students to achieve holistic development of children through the construction of positive challenges leading to positive movement experiences for all.

## Data Availability

The datasets presented in this article are not readily available because the data that support the findings of this study are not available due to ethical restrictions. Requests to access the datasets should be directed to natalie.houser@umanitoba.ca.
